# Statin-Induced Rhabdomyolysis Associated With Transjugular Intrahepatic Portosystemic Shunt Placement

**DOI:** 10.14309/crj.0000000000000774

**Published:** 2022-05-04

**Authors:** Eric C. Swei, Anantnoor K. Brar, Jonathan D. Rice, Ike I. Kim, Virginia M. Knez, Christopher F. Doe, Lisa M. Forman

**Affiliations:** 1 Division of Gastroenterology, University of Colorado School of Medicine, Aurora, CO; 2 Division of Internal Medicine, University of Colorado School of Medicine, Aurora, CO; 3 Skaggs School of Pharmacy and Pharmaceutical Sciences, University of Colorado, Aurora, CO; 4 Division of Pathology, University of Colorado School of Medicine, Aurora, CO; 5 Division of Vascular and Interventional Radiology, University of Colorado School of Medicine, Aurora, CO

## Abstract

Rhabdomyolysis is a known rare and potentially lethal complication of statin use. This toxic effect is potentiated by alterations in hepatic physiology in patients with cirrhosis. Transjugular intrahepatic portosystemic shunt placement has the potential to further compound this effect; yet, examples of this have not previously been described in the literature. We present a case of a patient who experienced statin-induced rhabdomyolysis likely as a direct consequence of transjugular intrahepatic portosystemic shunt placement.

## INTRODUCTION

Patients with cirrhosis are subject to physiological alterations that influence drug metabolism including impairment of metabolizing enzymes such as CYP450, decreased plasma protein synthesis, and alterations in splanchnic blood flow that increase drug bioavailability.^1^ Transjugular intrahepatic portosystemic shunt (TIPS), which is increasingly used to treat complications of cirrhosis, compounds this effect by further altering splanchnic hemodynamics.^2^ Despite a number of reports describing toxicity of various drugs after TIPS placement,^1–6^ clinicians may not intuitively recognize when potentially harmful medications should be dose-adjusted after placement. We present the case of a patient who experienced statin-induced rhabdomyolysis likely as a direct result of TIPS placement.

## CASE REPORT

**Figure 1.**
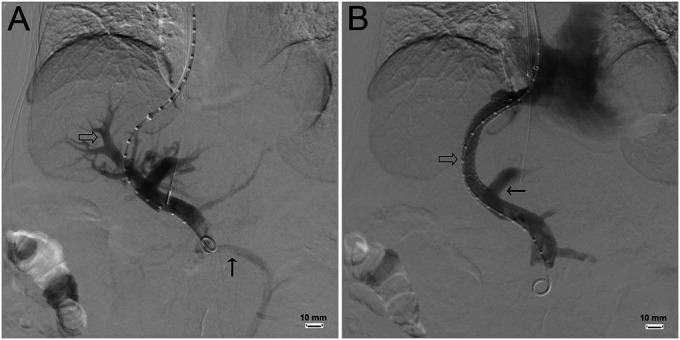
(A) Before TIPS placement, portal vein injection shows flow to the intrahepatic portal vein branches (open arrow). Incidental note is made of hepatofugal flow in the inferior mesenteric vein (solid arrow). (B) After TIPS placement, portal vein injection shows absent intrahepatic portal flow, with nearly all flow going through the TIPS (open arrow) and into the right atrium. Minimal flow is seen in the left portal vein (solid arrow). TIPS, transjugular intrahepatic portosystemic shunt.

A 68-year-old Asian woman with a history of nonalcoholic steatohepatitis cirrhosis, chronic kidney disease, and multiple previous cerebrovascular accidents underwent elective TIPS placement for refractory ascites (Figure 1) using intravenous propofol for sedation and without any notable postprocedural sequelae. At the time of postprocedure discharge, her liver function tests were notable only for a mildly elevated alkaline phosphatase (181 U/L, reference range 39–117 U/L), alanine aminotransferase (ALT 52 U/L, reference range 7–52 U/L), and aspartate aminotransferase (AST 36 U/L, reference range 12–39 U/L). Over the subsequent 2 weeks, she developed progressive lower extremity edema and weakness, prompting readmission. On presentation, she was noted to have bilateral pitting edema to the thighs, stable vital signs, and proximal muscle weakness with a nonfocal neurological examination. Laboratory test results were notable for a marked increase in alkaline phosphatase (315 U/L), ALT (103 U/L), AST (366 U/L), indirect bilirubin (2.0 mg/dL, reference range 0–1.0 mg/dL), total bilirubin (2.8 mg/dL, reference range 0.1–1.3 mg/dL), and internationalized normal ratio (1.8). Creatinine was 2.32 mg/dL, which was her baseline. Owing to proximal muscle weakness and the elevation in AST, there was clinical concern for rhabdomyolysis. Urinalysis demonstrated a large amount of blood but low red blood cell content, suggestive of myoglobinuria. Creatinine phosphokinase was elevated at 22,080 U/L (reference range 30–223 U/L), confirming the diagnosis of rhabdomyolysis.

**Figure 2.**
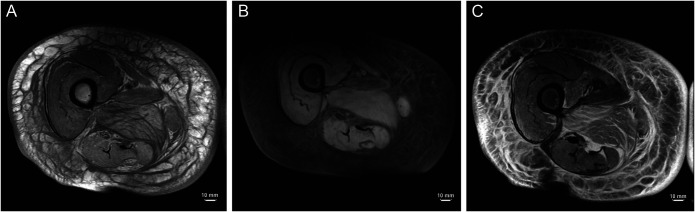
(A) Precontrast and (B) postcontrast axial T1 fat-saturated image of the right thigh. There is minimal change in the intrinsic signal of the musculature from precontrast to postcontrast images, indicative of poor enhancement. (C) Axial T2 fat-saturated image of the same thigh showing diffuse high-intensity signal, most substantially involving the distal adductor and proximal vastus muscles, indicative of intramuscular edema. Together, these changes are consistent with an inflammatory myositis.

**Figure 3.**
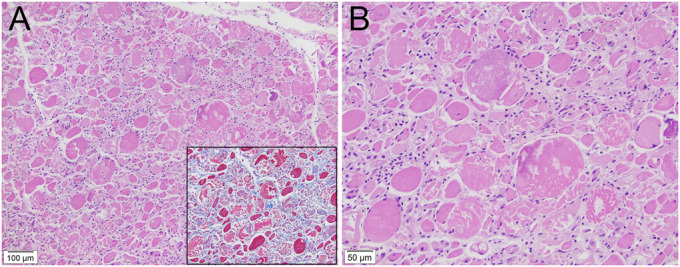
Photomicrographs of the rectus femoris muscle. (A) Hematoxylin and eosin (H&E) stain shows active myopathic damage, necrosis, and myofiber atrophy without significant inflammation, inclusion bodies, or evidence of vasculitis (10× objective). Inset with special stain trichrome shows no significant fibrosis (20× objective) nor were other signs of chronicity seen, indicating the changes were acute. (B) Higher magnification of H&E confirms the above findings (20× objective).

An MRI of the thighs was obtained to rule out myositis as an inciting cause of her rhabdomyolysis and in fact was initially suggestive of an inflammatory myositis (Figure 2). However, an autoimmune myositis panel was negative, and a muscle biopsy was performed, which showed histological findings of acute necrotizing myopathy without a significant inflammatory component (Figure 3). Drug-induced myopathy was thus suspected as the primary diagnosis. Chart review demonstrated that the patient had been on long-standing high-intensity statin therapy (atorvastatin 80 mg) for secondary prevention since 8 years before presentation when she suffered a cerebrovascular accident despite moderate-dose statin therapy (simvastatin 40 mg).

The sudden development of statin-induced rhabdomyolysis despite a stable dose was attributed to alterations in hepatic blood flow caused by recent TIPS placement that resulted in increased drug bioavailability (Figure 1); therefore, the medication was stopped. Four days after stopping her medication, the creatinine phosphokinase peaked at 92,250 with liver enzymes peaking as well (AST 1,717 U/L and ALT 706 U/L). Despite supportive care and withdrawal of the offending medication, progressive renal failure developed with anuria with creatinine peak of 4.99 mg/dL. Despite a time-limited trial of dialysis, she ultimately expired.

## DISCUSSION

Although the exact mechanism for statin-induced rhabdomyolysis remains a topic of debate, it is clear that the risk of developing this condition increases in a dose-dependent manner^7^ exacerbated by both cirrhosis and TIPS placement. Our patient may have been particularly vulnerable to this phenomenon, given that she had multiple risk factors known to be associated with the development of statin toxicity (female sex, Asian ethnicity, multisystem disease, particularly including renal insufficiency, and generalized frailty).^8^ Furthermore, despite having been previously exposed to propofol multiple times without sequelae, we note that propofol exposure has been associated with the propagation of statin toxicity and may have contributed in the context of TIPS placement.^9^

Regarding specific sequelae of cirrhosis and TIPS placement that can contribute to statin toxicity, patients with cirrhosis have decreased first-pass hepatic metabolism, decreased protein binding, and decreased drug clearance, which leads to increased plasma drug concentrations.^10^ Atorvastatin and rosuvastatin, 2 of the most commonly prescribed statins, undergo extensive first-pass metabolism by hepatic CYP3A4 and CYP2C9, respectively, and thus are subject to significantly increased bioavailability in patients with cirrhosis.^10,11^ Although TIPS placement restores portal venous flow in patients with cirrhosis, it further alters drug bioavailability through increased splanchnic blood flow and intrahepatic shunting.^12^ Increased bioavailability and decreased clearance of drugs that have significant first-pass metabolisms such as bisoprolol, nifedipine, budesonide, midazolam, and phenytoin^1,3,4,6^ have been demonstrated after TIPS. However, knowledge of the exact effects of TIPS on statin metabolism is not well understood. Failure to account for this effect can lead to increased risk of adverse drug reactions, as seen in this case where despite remaining on a stable statin dose, the patient developed rhabdomyolysis soon after TIPS creation.

Given statins are the most commonly prescribed cholesterol-lowering medications in the United States and have a crucial role in the treatment of nonalcoholic steatohepatitis, there is a need for clinicians to understand statin pharmacokinetics after TIPS. Although the ideal statin type and dose after TIPS is unknown, a cautious step-up approach with a low dose of a hydrophilic statin, such as pravastatin, with close monitoring may protect from serious adverse events.^13^ As this case highlights, increased awareness and thoughtful postprocedural medication reconciliation is crucial to prevent harm.

## DISCLOSURES

Author contributions: EC Swei and AK Brar wrote and approved the manuscript. EC Swei, AK Brar, JD Rice, and LM Forman edited the manuscript and revised the manuscript for intellectual content. EC Swei and AK Brar reviewed the literature. CF Doe and VM Knez provided the images. EC Swei is the article guarantor.

Financial disclosures: None to report.

Informed consent could not be obtained for this case report. All identifying information has been removed.

## References

[1] Weersink RA Bouma M Burger DM . Evaluating the safety and dosing of drugs in patients with liver cirrhosis by literature review and expert opinion. BMJ Open. 2016;6:e012991. 10.1136/bmjopen-2016-012991 PMC5073492 27733414

[2] Rodríguez-Laiz JM Bańares R Echenagusia A . Effects of transjugular intrahepatic portasystemic shunt (TIPS) on splanchnic and systemic hemodynamics, and hepatic function in patients with portal hypertension. Dig Dis Sci. 1995;40:2121–7. 7587778 10.1007/BF02208995

[3] Chalasani N Gorski JC Patel NH . Hepatic and intestinal cytochrome P450 3A activity in cirrhosis: Effects of transjugular intrahepatic portosystemic shunts. Hepatology. 2001;34:1103–8. 11731998 10.1053/jhep.2001.29306

[4] Bassell T Aminlari A Hayden S . Phenytoin toxicity after transjugular intrahepatic portosystemic shunt (TIPS). J Emerg Med. 2021;60:54–7.e1. 33160822 10.1016/j.jemermed.2020.09.010

[5] Spriet I Meyfroidt G Maleux G . The impact of a transjugular intrahepatic portosystemic shunt on the pharmacokinetics of caspofungin in a critically ill patient. Pharmacology. 2012;90:247–50. 23007631 10.1159/000342906

[6] Jayakrishnan T Babu M Goodnow S . Budesonide-induced hyperosmolar hyperglycemic state following transjugular intrahepatic portosystemic shunt. AACE Clin Case Rep. 2020;6:e265–8. 32984535 10.4158/ACCR-2020-0216 PMC7511094

[7] Golomb BA Evans MA. Statin adverse effects: A review of the literature and evidence for a mitochondrial mechanism. Am J Cardiovasc Drugs. 2008;8:373–418. 19159124 10.2165/0129784-200808060-00004 PMC2849981

[8] Ward NC Watts GF Eckel RH. Statin toxicity: Mechanistic insights and clinical implications. Circ Res. 2019;124:328–50. 31170055 10.1161/CIRCRESAHA.119.315233

[9] Francis L Bonilla E Soforo E . Fatal toxic myopathy attributed to propofol, methylprednisolone, and cyclosporine after prior exposure to colchicine and simvastatin. Clin Rheumatol. 2008;27:129–31. 17628739 10.1007/s10067-007-0696-9

[10] Sung S Al-Karaghouli M Kalainy S . A systematic review on pharmacokinetics, cardiovascular outcomes and safety profiles of statins in cirrhosis. BMC Gastroenterol. 2021;21:120. 33726685 10.1186/s12876-021-01704-w PMC7967963

[11] Kane SP. ClinCalc DrugStats Database. Volume 2021. Version 2021. 10 ed, 2021.

[12] Rostami-Hodjegan A Tucker GT. The effects of portal shunts on intestinal cytochrome P450 3A activity. Hepatology 2002;35:1549–50; author reply 1550–1. 12029646 10.1053/jhep.2002.33215

[13] Vargas JI Arrese M Shah VH . Use of statins in patients with chronic liver disease and cirrhosis: Current views and prospects. Curr Gastroenterol Rep. 2017;19:43. 28752475 10.1007/s11894-017-0584-7 PMC5822686

